# Identification of closely related *Ixodes* species by protein profiling with MALDI-TOF mass spectrometry

**DOI:** 10.1371/journal.pone.0223735

**Published:** 2019-10-17

**Authors:** Pierre H. Boyer, Lionel Almeras, Olivier Plantard, Antoine Grillon, Émilie Talagrand-Reboul, Karen McCoy, Benoît Jaulhac, Nathalie Boulanger

**Affiliations:** 1 EA 7290: Early Bacterial Virulence: *Borrelia* Group, CHRU Strasbourg, Fédération de Médecine Translationnelle, Strasbourg, France; 2 Unité Parasitologie et Entomologie, Département Microbiologie et maladies infectieuses, Institut de Recherche Biomédicale des Armées, Marseille, France; 3 Aix Marseille Univ, IRD, SSA, AP-HM, VITROME, Marseille, France; 4 IHU Méditerranée Infection, Marseille, France; 5 BIOEPAR, INRA, Oniris, Université Bretagne Loire, Nantes, France; 6 Maladies Infectieuses & Vecteurs: Ecologie, Génétique, Evolution & Contrôle (MIVEGEC), Université de Montpellier–CNRS—IRD, Centre IRD, Montpellier, France; 7 French National Reference Center for *Borrelia*, Hôpitaux Universitaires de Strasbourg, Strasbourg, France; University of Minnesota, UNITED STATES

## Abstract

Ticks are vectors of infectious diseases of major importance in human and veterinary medicine. For epidemiological studies, accurate identification of ticks is crucial to define their potential role as vectors and to develop control and prevention strategies. Although morphological and molecular methods are widely used to identify ticks, an innovative approach using MALDI-TOF MS technology recently emerged as an alternative tool. Previous works showed that MALDI-TOF MS was highly effective in identifying ticks, but these works mainly tested tick specimens of different genera. To confirm the accuracy of this new tool for tick identification, nine closely related tick species belonging to the *Ixodes* genus were analysed, specimens of the *Dermacentor reticulatus* species were also included in the analysis as an outer group. Three of the species used for the present study belonged to the *I*. *ricinus* species complex, which are known to transmit *Borrelia burgdorferi* sensu lato, the causative agent of Lyme borreliosis. A total of 246 tick specimens were submitted to MALDI-TOF MS analysis, and two body parts (half-idiosoma and four legs) were individually investigated. For each body part, intraspecies reproducibility and interspecies specificity of the MS profiles were determined. The profile analysis revealed that the main determinant for spectra clustering was the tick species for both legs and half-idiosoma. For each body part, a reference database of spectra was set up including 2 to 5 specimens per species randomly selected, and genotyped using 16s rDNA and COI genes to confirm their morphological identification. Both created spectral databases were individually blind tested with their respective body part using the remaining specimens, which were correctly identified in 98.5% of the cases. MALDI-TOF MS is a reliable tool for tick identification, including specimens belonging to closely related species and hardly distinguishable using morphology. The 4-legs as well as the half-idiosoma of ticks can now be applied for specimen identification using two different databases. The combined use of these two body parts improves the rate of tick identification and their confidence level.

## Introduction

Ticks are obligate hematophagous ectoparasites feeding on vertebrate hosts. Although wild and domestic animals are the primary source of tick blood meals, humans can be accidental hosts and are susceptible to several tick-borne diseases (TBDs) [1]. During blood meals of infective ticks, bacteria, viruses, and parasites can be transmitted to vertebrate hosts. Among TBDs affecting humans, Lyme borreliosis is the most prevalent in the Northern Hemisphere [2]. Lyme borreliosis presents as cutaneous (erythema migrans, acrodermatitis, lymphocytoma), articular (Lyme arthritis), and neurological (neuroborreliosis) symptoms. Spirochetes belonging to the *Borrelia burgdorferi* sensu lato (sl) group are the causative agents of Lyme borreliosis and are transmitted by ticks belonging to the *Ixodes* genus [2].

Approximately 244 species are currently part of the *Ixodes* genus [3], but 29 species are recognized as potential vectors of human diseases [4] and only a few are proven vectors of *B*. *burgdorferi* sl. [5,6]. The main vectors of human Lyme borreliosis are *I*. *ricinus* and *I*. *persulcatus* in Eurasia, and *I*. *scapularis* and *I*. *pacificus* in North America [2]. Accurate identification of tick species is initially required to evaluate the risk of tick bite exposure and to implement vector control measures [7,8]. Until recently, methods based on morphology and DNA sequencing were the two cornerstones of tick identification [9]. Morphological identification is based on the use of taxonomic criteria included in dichotomous keys [10]. However, this identification tool has several drawbacks [9] as it is time-consuming and as correct morphological identification relies on entomological expertise [11] and specimen integrity. Intraspecific morphological variation can also prevent reliable identification, particularly when using decisive criteria. This phenomenon is particularly frequent with immature stages of ticks (*i*.*e*., larvae or nymphs) [12], which are very often collected at these developmental stages.

To overcome the limitations of morphological identification of ticks, molecular identification techniques mainly based on gene amplification and DNA sequencing have increasingly been used over the last decade [13]. The most frequently targeted genes have a mitochondrial origin (*e*.*g*., 12S, 16S ribosomal DNA, cytochrome oxidase subunit 1), but nuclear genes are also used (18S ribosomal DNA, internal transcribed spacers 1 or 2) [14]. Despite the effectiveness and accuracy of this approach, no consensus has been reached on selecting a single genetic marker to identify tick species. No universal primers allow for the amplification of a given gene in all species [13]. Finally, the GenBank database is incomplete for some species and can be inaccurate for others (*i*.*e*., wrong initial identification). Moreover, molecular biology techniques remain time-consuming and require expensive reagents. Their use is therefore limited to tick monitoring on a large scale.

An alternative approach for arthropod identification, based on protein profiling analysis, has recently been developed [9]. Using matrix-assisted laser desorption-ionization time-of-flight mass spectrometry (MALDI-TOF MS), unknown specimen identification is performed by comparing the MS spectrum of these specimens − representing a fingerprint of the arthropod’s most abundant proteins − with reference MS spectra database of known species. The accuracy of specimen identification relies on how well the MS spectrum matches the spectrum of a known species. This proteomic tool has already been used for the identification of several arthropod families such as biting midges, fleas, sand flies, mosquitoes, and ticks [9]. This fast and reliable method is now emerging for the identification of arthropods [15,16]. Nevertheless, several factors such as storing mode (alcohol-preserved or fresh specimens), engorgement status [17], or standardization method of samples can alter the reproducibility of species-specific MS spectrum protein profiles [18]. Standardization and automatization of protocols increase the intraspecies reproducibility and interspecies specificity of MS profiles [19,20]. The development of this optimized protocol for sample preparation underlined the importance of upgrading the reference MS spectra database with samples processed under the same conditions. Moreover, it was recently demonstrated that legs and half-idiosoma of *I*. *ricinus* generated specific MS spectra [21]. Each of these two body parts can be used for tick identification by MS, which could improve the accuracy of specimen identification despite slight variations observed according to environmental or spatiotemporal conditions [22].

Based on enhanced preparation guidelines, the present study aimed to create and validate a primary MS spectra reference database for the identification of closely related tick species of the *Ixodes* genus using legs and half-idiosoma. A total of 10 distinct tick species were selected including nine tick species of the *Ixodes* genus among which three species belonged to the *I*. *ricinus* complex [23] (*I*. *ricinus*, *I*. *persulcatus*, and *I*. *scapularis*). Ticks of this complex play a key role in the transmission of spirochetes of the *B*. *burgdorferi* sl complex to humans [2] and several other TBDs such as *Anaplasma phagocytophilum* infections, *Babesia* spp. infections, and tick-borne encephalitis virus infections [10]. The added value of this MS reference database for rapid and accurate entomological diagnosis of tick specimens is here discussed in the context of TBDs.Materials and methods.

### Ticks sampling and morphological identification

Adult and nymphal ticks were either laboratory reared or collected in the field (on or off the vertebrate host). Laboratory-reared ticks were maintained in climatic chambers (25°C, with a relative humidity of 80–90%) and successive generations were obtained by feeding the ticks. Wild caught ticks were either collected by dragging a white flannel flag (1x1 m) over low vegetation, or were sampled from the host animals. Ticks were sampled in several countries and were sent alive at room temperature or frozen. The stage and sex of the collected ticks were determined by morphological identification under a binocular microscope at a magnification of ×56 (Leica M80, Leica, Nanterre, France) using standard taxonomic keys [24–26]. For *I*. *ricinus* and *D*. *reticulatus* specimens were collected at different geographic places and different months of the year (Table 1).

**Table 1:** Details of tick collection classified by species.

	Geographical origin	Number of specimens	Tick stages (Adult (sex^#^)/Nymph/Larva)	Number of specimens engorged
*I*.* ventalloi*	Nantes region, France	17	15 (8M/7F)/ 2 / 0	7
*I*.* ricinus*	Alsace & Ain, France	109	13 (6M/7F) / 93 / 3	14
*I*.* persulcatus*	Riga, Latvia	18	14 (9M/5F) / 4 / 0	0
*I*.* scapularis*	Rhode Island, USA	27	27 (10M/17F) / 0 / 0	0
*I*.* acuminatus*	Nantes region, France	13	4 (0M/4F) / 9 / 0	0
*I*.* uriae*	Hornøya, Norway	13	8 (4M/4F) / 5 / 0	0
*I*.* vespertilionis*	Nantes region, France	4	4 (2M/2F) / 0 / 0	0
*I*.* hexagonus*	Nantes region, France	28	16 (0M/16F) / 12 / 0	28
*I*.* frontalis*	Nantes region, France	4	2 (0M/2F) / 2 / 0	4
*D*.* reticulatus*	Alsace & Ain, France	13	13 (7M/6F) / 0 / 0	0
**Total**		246	116 (40M/76F)/127/3	53

^#^M (male), F (female).

### Tick dissection and sample preparation

Each tick was rinsed once with 70% (v/v) ethanol then twice with distilled water as previously described [27]. After drying, the specimen was dissected with a sterile surgical blade. Four legs were removed and the idiosoma was longitudinally cut in two equal parts. Legs and the half-idiosoma were used independently for MALDI-TOF MS analyses.

### DNA extraction

DNA of each half idiosoma with legs was individually extracted with ammonium hydroxide (Sigma-Aldrich) as previously described [28,29]. Purified DNA from each tick was stored at -80°C until use.

### Molecular identification of ticks

To confirm morphological identification, all the specimens included in the database were genotyped using the COI gene and 16s rDNA gene. For the COI gene, Cox1F (5’–GGAACAATATATTTAATTTTTGG–3’) and Cox1R (5’–ATCTATCCCTACTGTAAATATATG–3’) [13] were used as forward and reverse primers respectively, the predicted size of the product was around 800 bps. For the 16s rDNA a fragment with a predicted size of 400 bps was amplified by PCR, using 5’–CCGGTCTGAACTCAGATCAAGT–3’ as the forward primer and 5’–GCTCAATGATTTTTTAAATTGCTGT–3’ as the reverse one [30]. PCR amplifications were performed on GenAmp PCR system 9700 (Applied Biosystems, Courtaboeuf, France) using a HotStartTaq (Qiagen, Les Ulis, France). The PCR program for the COI amplification included an initial denaturation step of 15 min at 94°C, followed by 10 cycles of denaturation at 92°C for 1 min, annealing at 42°C for 1 min, and elongation at 72°C for 1 min 30 s, followed by 32 cycles of denaturation at 92°C for 1 min, annealing at 46°C for 35 s, and elongation at 72°C for 1 min 30 s, followed by a final elongation at 72°C for 7 min. For the 16s rDNA, the protocol was: 15 min initial denaturation at 94°C, followed by 7 cycles of denaturation at 92°C for 30 s, annealing for 30s with an annealing temperature increased by 0.3°C every second cycle from 47 to 48.8°C, elongation for 45s at 72°C, followed by 28 cycles with an annealing temperature of 50°C and finally a 7 min extension step at 72°C. The success of the PCR amplification was checked by performing agarose gel electrophoresis. After purification, amplicons were sequenced with the primer used for amplification on an ABI 3730 XL system (Applied Biosystems, Foster City, CA, USA), using the BigDye® Terminator v3.1 Cycle Sequencing Kit (Life Technologies, Carlsbad, CA, USA) as previously described [31]. Quality of sequences was assessed by inspecting the chromatogram with SeqTrace [32], then the forward and reverse sequences were assembled and converted into high-quality finished DNA sequences using the SeqTrace software [32]. The sequences were compared with sequences from GenBank (http://blast.ncbi.nlm.nih.gov).

### Phylogenetic analyses

After gene sequences alignment with the Clustal ω2 algorithm in the MEGA 7.0 software, two maximum likelihood trees based on the 16s rDNA or the COI gene were constructed using the MEGA 7.0 software. The most appropriate model was determined with the modified Akaike criterion calculated with IQ-TREE tool available at http://iqtree.cibiv.univie.ac.at. The general time reversible model, with gamma distributed rate variation across sites and a proportion of invariable sites, was selected for the phylogenetic analysis. Support for internal nodes was estimated using the nonparametric bootstrap method with 100 replications.

### MALDI-TOF MS analyses

#### Sample preparation

The four legs and half-idiosoma of each tick specimen were homogenized separately using an automated grinding method, the FastPrep-24 device (MP Biomedicals, Illkirch-Graffenstaden, France) with a small amount of glass beads (with a diameter ≤106 μm) (Sigma, Lyon, France). The device settings were identical to those previously established as optimal for ticks [20]. For tick legs, a mix of 20 μL of 70% (v/v) formic acid (Sigma) plus 20 μL of 50% (v/v) acetonitrile (Fluka, Buchs, Switzerland) was used. For half-idiosoma, 30 μL of 70% (v/v) formic acid (Sigma) and 30 μL of 50% (v/v) acetonitrile were used.

After sample homogenization, a quick centrifugation at 200 g for 1 min was done to pellet debris, and 1 μL of the supernatant of each sample was spotted on the MALDI-TOF steel target plate in quadruplicate (Bruker Daltonics, Wissembourg, France). After drying, each spot was coated with 1 μL of matrix solution composed of saturated α-cyano-4-hydroxycynnamic acid (Sigma, Lyon, France), 50% acetonitrile (v/v), 2.5% trifluoroacetic acid (v/v) (Aldrich, Dorset, UK) and HPLC-grade water. The target plate was then air-dried for a few minutes at room temperature prior to being introduced in the Microflex LT MALDI-TOF Mass Spectrometer (Bruker Daltonics) for analysis. To control matrix quality, sample loading, and MALDI-TOF apparatus performance, the matrix solution was deposited in duplicate onto each MALDI-TOF plate with and without bacterial control (*Pseudomonas aeruginosa* ATCC 27853).

#### MALDI-TOF MS parameters

Protein mass profiles of each tick body part were generated using a Microflex LT MALDI-TOF Mass Spectrometer (Bruker Daltonics, Germany), with detection in the linear positive-ion mode at a laser frequency of 50 Hz within a mass range of 2–20 kDa. The acceleration voltage was 20 kV, and the extraction delay time was 200 ns. Each spectrum corresponded to ions obtained from 240 laser shots performed in six regions of the same spot and automatically acquired using the AutoXecute method with the default parameters of the flexControl v3.4 software (Bruker Daltonics). The spectrum profiles were visualized with flexAnalysis v3.4 software, MALDI biotyper Compass Explorer v4.1.70 (Bruker Daltonics, Germany) and ClinProTools v3.0 software (Bruker Daltonics) for data processing.

#### Spectra analyses

The MS spectra resulting from automatic protocols were first visually controlled by the flexAnalysis v3.4 software. Then, to assess intra-species reproducibility by body part, spectra were loaded on ClinProTools v3.0 software. Next, the MS profile specificity was assessed, using the following method. The four spectra of two to five specimens per species underwent an MSP (Main Spectra Projection) processing using the manufacturer’s method. Cluster analysis using the MSP dendrogram function of MALDI biotyper Compass Explorer v4.1.70 software was performed. Briefly, it is based on the comparison between the MSP given by the MALDI-Biotyper software and clustered according to protein mass profile (i.e., their mass signals and intensities) and the resulting MS dendrogram illustrating how samples are related to each other. The reproducibility and the specificity of the MS profiles according to the body part per species were also assessed based on a Principal Component Analysis (PCA). The PCA tool of the ClinProTools software was used with the manufacturer settings. The composite correlation index (CCI) tool from MALDI biotyper Compass Explorer software was used to assess the spectral variations within and between each sample group, according to the body part. Correlation values (expressed as the mean ± standard deviation, SD) reflecting reproducibility for the MS spectra, were used to estimate MS spectra distance between species for each body part.

#### Reference database creation

Based on the consistency of the morphological and molecular results of tick identification, two to five specimens per species and body part were used to create reference MS spectra database (S1 File). Legs and half-idiosoma from each tick species exhibiting reproducible and specific MS spectra were then included in a MS spectra reference database. To create the database, MSP reference spectra were included using spectra from two to five specimens per species. Average spectra (MSP, Main Spectrum Profile) were created by combining the four spectra of one tested sample, using the automated function of the MALDI-Biotyper software (Bruker Daltonics). MSP were created on the basis of an unbiased algorithm using peak position, intensity and frequency data using the default parameter set of the “Bio Typer MSP Creation Standard Method”. Briefly, the maximum mass error of each single spectrum was 2000 Da, the desired mass error for the MSP was 200 Da, the desired peak frequency minimum was 25% and the maximum desired peak number for the MSP was 70.

#### Assignment of discriminating peaks

To assign discriminating peaks according to tick species by body-part, MS spectra from each species and both body-parts were imported into ClinProTools software. The software was used to generate a peak list for each species per body-part in the 2 to 20 kDa mass range and to identify discriminating peaks. The settings in ClinProTools software for spectrum preparation were the following: a resolution of 300; a noise threshold of 2.00; a maximum peak shift of 800 ppm and a match to calibrating agent peaks of 10%. Peak calculation and selection were performed on individual spectrum with a signal-to-noise threshold of 2.00 and an aggregation of 800 ppm. The spectra were then analysed using the genetic algorithm (GA) model using the default parameters, which displays a list of discriminating peaks. The maximum number of peaks in the model was set to 150 the maximum number of generations was set to 250 and the number of neighbours was five for K nearest neighbours (KNN) classification. Manual inspection and validation of the selected peaks by the operator gave a recognition capability (RC) value together with the highest cross-validation (CV) value. The presence or absence of all discriminating peaks generated by the GA model was controlled by comparing the average spectrum of each species per body-part.

#### Blind tests

A blind test was performed with the remaining tick specimens not included in the reference MS spectra databases. A total of 808 and 624 MS spectra from tick legs and half-idiosoma were tested against their respective reference spectra database. The reliability of tick species identifications was estimated using the log score values (LSVs) obtained from the MALDI-Biotyper software, which ranged from 0 to 3. These LSVs correspond to the degree of similarity between the MS reference spectra in the database and those submitted by blind tests. A LSV was obtained for each spectrum of the samples tested. According to previous studies [19,20], an LSV of at least 1.8 should be obtained to be considered reliable for species identification. As proposed by Kumsa et al. [33], an additional criterion of a 0.2 minimum difference between the score of the best species match and the second species match score was required. To test the specificity of the generated MS profiles, all spectra were queried against the commercial bacteria database (Bruker Daltonics) including MSPs from new bacterial species or strains found in the laboratory (library of 7393 MSPs, database from November, 8, 2017) using MALDI biotyper Compass Explorer v4.1.70 software.

### Ethical statement

The protocols to maintain tick colony (N°APAFIS 886–2015062209279407) and for blood feeding of wild ticks (N°APAFIS 6040–2016111411067314) were approved by the Comité Régional d’Ethique en Matiére d’Expérimentation Animale de Strasbourg (CREMEAS—Committee on the Ethics of Animal Experiments of the University of Strasbourg). Ethical approval of the collection of *I*. *uriae* from seabirds was obtained from the Norwegian National Food and Safety Authories (ID 8947) and the Finnmark county government (Fylkesmannen). The authority who issued the permission to collect ticks from public locations was the ONF (Office National des forêts, France). Privately owned areas were sampled after agreement with the owners. Ticks were not collected from endangered or protected species except hedgehogs. Hedgehogs (on which *I*. *hexagonus* specimens were sampled) and blackbirds (on which *I*. *frontalis* specimens were sampled) were brought by civilians to Centre Vétérinaire de la Faune Sauvage et des Ecosystèmes des Pays de la Loire a wildlife health centre Near Nantes. To grant animals an easy and complete recovery, all ectoparasites are removed on arrival at the center as a standard procedure.

All the protocols listed above follow the European directive 2010/63/EU and were performed in animal facilities N° A67-482-34.

## Results

### Morphological and molecular identification

A total of 246 ticks were included in the present study. Ticks were collected from the field (n = 174), on animals (n = 57), and from laboratory rearing colonies (n = 15). Morphological identification revealed that all specimens investigated belonged to nine different species of the *Ixodes* genus (*I*. *ventalloi*, *I*. *ricinus*, *I*. *persulcatus*, *I*. *scapularis*, *I*. *acuminatus*, *I*. *uriae*, *I*. *vespertilionis*, *I*. *hexagonus*, *I*. *frontalis*), except for 13 specimens of *Dermacentor reticulatus* ticks. Data on tick sampling, including species, sex type, developmental stage, and origin, are summarized in Table 1 and S1 Table.

**Fig 1:**
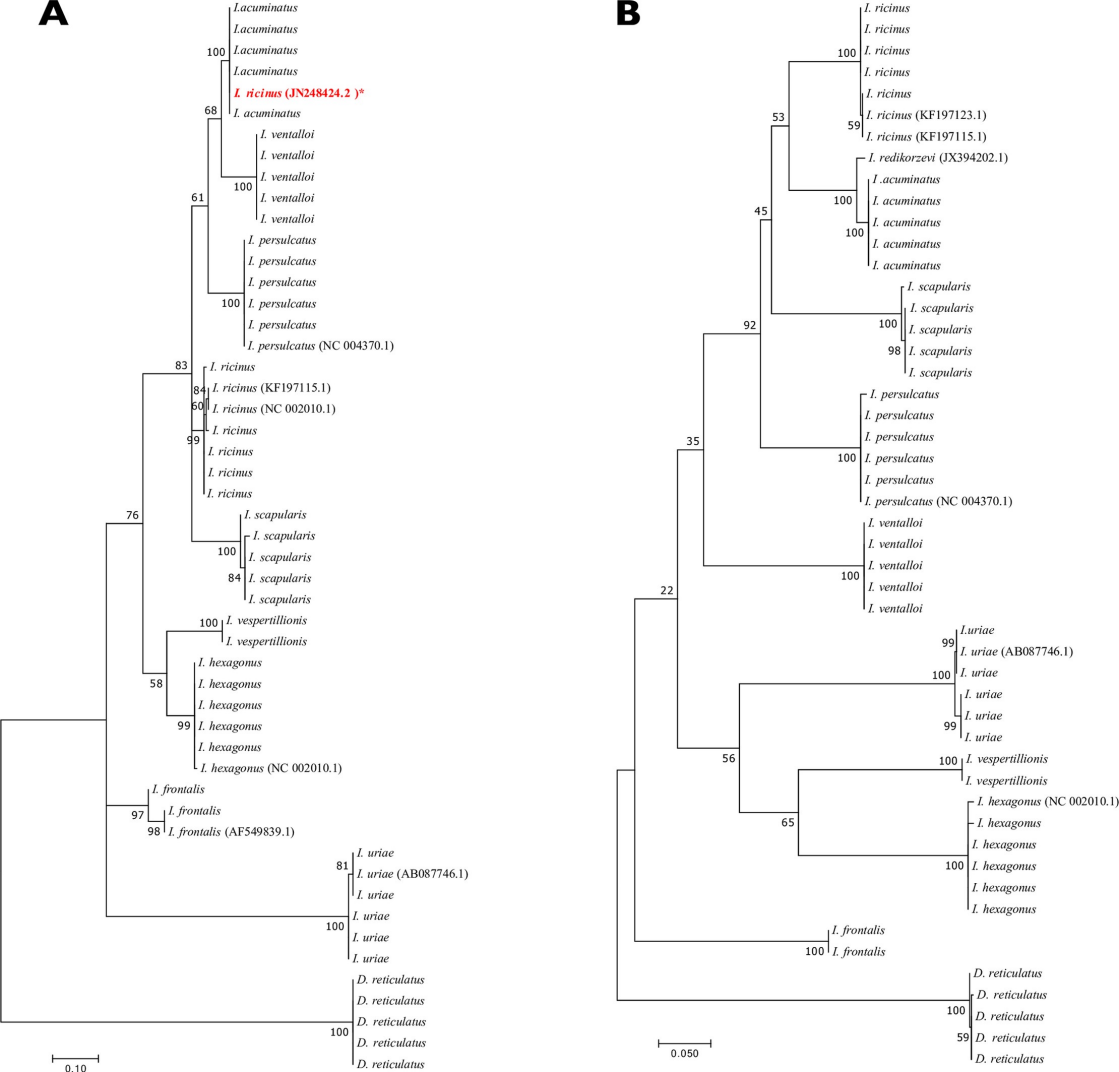
**Unrooted maximum–likelihood trees based on the sequences of the 16s rDNA gene (A) and the COI gene (B) of the 44 specimens included in the database and GenBank sequences.** *A GenBank sequence attributed to *I*. *ricinus* (Accession number JN248424.2) clustered with *I*. *acuminatus* on the 16s rDNA gene tree.

To confirm morphological identification, 44 of the 246 specimens (2 to 5 specimens per species) were selected at random for molecular analysis. A GenBank query indicated that 16s rDNA and COI gene sequences were available for all species except for *I*. *acuminatus*. Sequencing and comparisons with GenBank database of the 16s rDNA gene and COI gene, using the BLAST functionality, revealed reliable and coherent tick species identification according to morphological data (Fig 1 and Table 2). Interestingly, using a BLAST analysis *I*. *acuminatus* COI sequence matched at 98% with a COI sequence of *I*. *redikorzevi*. Surprisingly, interrogating the GenBank database with 16s rDNA sequence obtained from the *I*. *acuminatus* specimens revealed 100% similarity with a single sequence of *I*. *ricinus* (Accession number JN248424.2). These results confirmed the reliability of morphological identifications. The sequences obtained for each species were submitted to the GenBank database, detailed accession numbers are summed up in the S2 Table.

**Table 2:** Details of the 44 specimens included in the reference database and homology to the reference sequences using BLAST.

Tick species	Tick stages (Adult (sex^#^)/Nymph)	Status	Origin	16s rDNA interrogation			COI interrogation		
				Identified species	% of identity	Accession number	Identified species	% of identity	Accession number
*D*. *reticulatus*	3F/2M/0N	Unengorged	Field	*D*. *reticulatus*	99–100%	KR870969.1-KX881100.1	*D*. *reticulatus*	99–100%	AF132829.1
*I*. *acuminatus*	2F/0M/3N	Unengorged	Lab breed	*I*.* ricinus*	100%	JN248424.2	*I*. *redikorzevi*	98%	JX394202.1
*I*. *frontalis*	1F/0M/1N	Engorged	Animal	*I*. *frontalis*	99–100%	KP769862.1	*I*. *frontalis*	99%	KU170492.1
*I*. *hexagonus*	2F/0M/3N	Engorged	Animal	*I*. *hexagonus*	99–100%	KJ414454.1-KP769862.1	*I*. *hexagonus*	99–100%	MG432679.1-AF081828.1
*I*.* persulcatus*	1F/4M/0N	Unengorged	Field	*I*.* persulcatus*	99–100%	KP283020.1	*I*.* persulcatus*	99–100%	AB073725.1
*I*.* ricinus*	2F/2M/1N	Unengorged	Field	*I*.* ricinus*	99–100%	AB819253.1-KP283020.1	*I*.* ricinus*	99–100%	KF197132.1-KF197134.1
*I*.* scapularis*	3F/2M/0N	Unengorged	Field	*I*.* scapularis*	99–100%	KF146643.1-KR092230.1	*I*.* scapularis*	100%	KC488301.1-KC488313.1
*I*. *uriae*	2F/0M/3N	Unengorged	Field	*I*. *uriae*	100%	AB087746.1-D88298.1	*I*. *uriae*	99–100%	AB087746.1-KX360345.1
*I*. *ventalloi*	0F/5M/0N	Unengorged	Field	*I*. *ventalloi*	100%	MG210720.1-KY231931.1	*I*. *ventalloi*	99%	KU178964.1
*I*.* vespertilionis*	1F/1M/0N	Unengorged	Field	*I*.* vespertilionis*	99%	KM455967.1	*I*.* vespertilionis*	99%	KR902758.1

^#^M (male), F (female)

### Reproducibility and specificity of MALDI-TOF MS spectra according to *Ixodes* tick species and body parts

**Fig 2:**
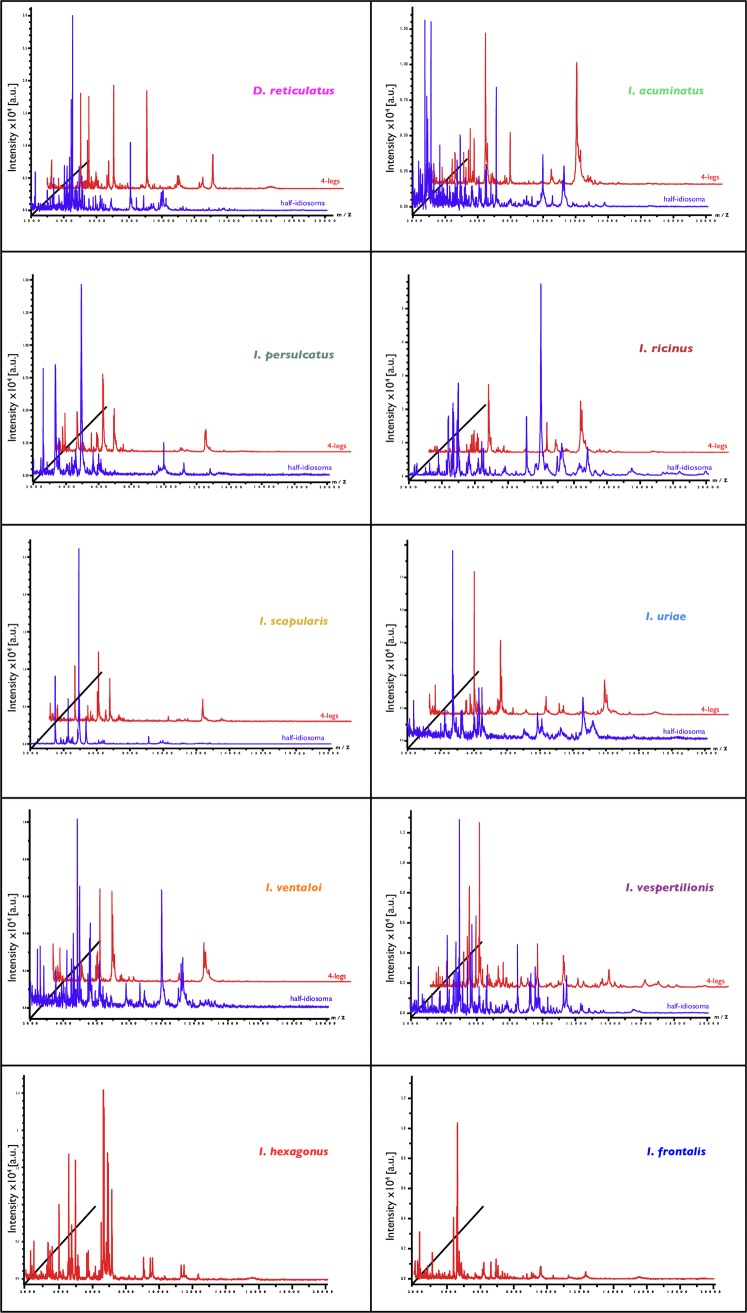
**Comparison of MALDI–TOF MS spectra from the four legs (in red) and half–idiosoma (in blue).** Representative MS spectra of legs and half–idiosoma of ticks, automatically standardized using FastPrep–24, are shown. Respective tick species and body parts are indicated on the right part of each spectrum. a.u., arbitrary units; m/z, mass–to–charge ratio.

**Fig 3:**
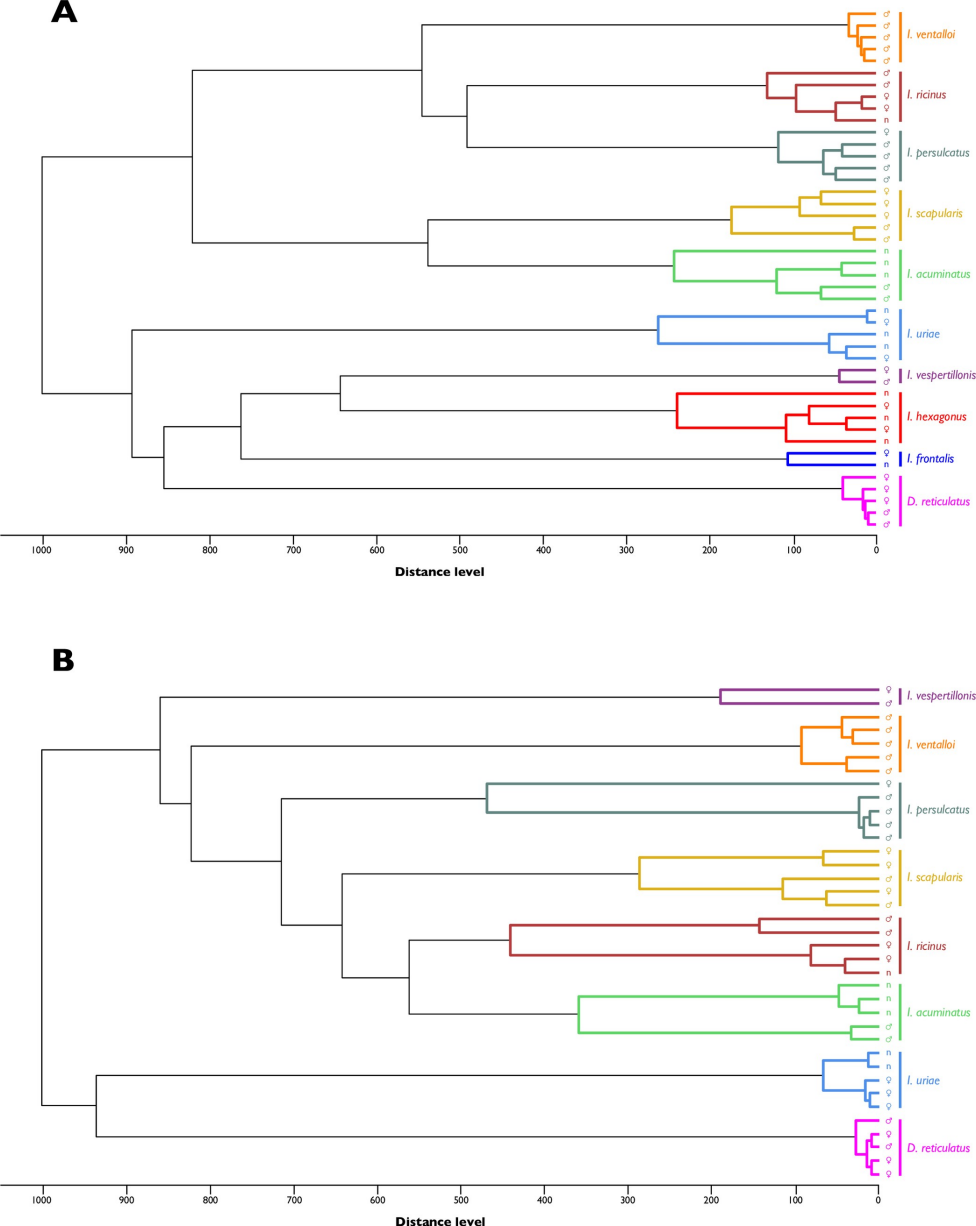
**MSP dendrograms of MALDI–TOF MS spectra from legs (A) and half–idiosoma (B) of ticks.** Two to five specimens per tick species were used to construct MSP dendrograms. Dendrograms were created using MALDI Biotyper Compass Explorer v4.1.40 software, and distance units represent the relative similarity of MS spectra. The same color code is used for each tick species. Genders of adult ticks are indicated by symbols and “n” corresponds to the nymphal stage.

To control the reproducibility and specificity of MS spectra according to tick species and body parts (44 legs and 37 half-idiosoma), the 44 specimens morphologically identified and confirmed by molecular biology technique were selected (Table 2). As all *I*. *hexagonus* and *I*. *frontalis* specimens were collected from hosts, they were all engorged. It has already been reported that blood contained in the tick’s gut interferes with MS spectra reproducibility and quality [17,27]. Only the legs of these two tick species were submitted to MS analysis. The MS spectra obtained were visually distinct between species and body parts (Fig 2). Clustering analyses of MS spectra from legs (Fig 3A) and half-idiosoma (Fig 3B) showed that all specimens of the same species gathered together on the same cluster.

**Fig 4:**
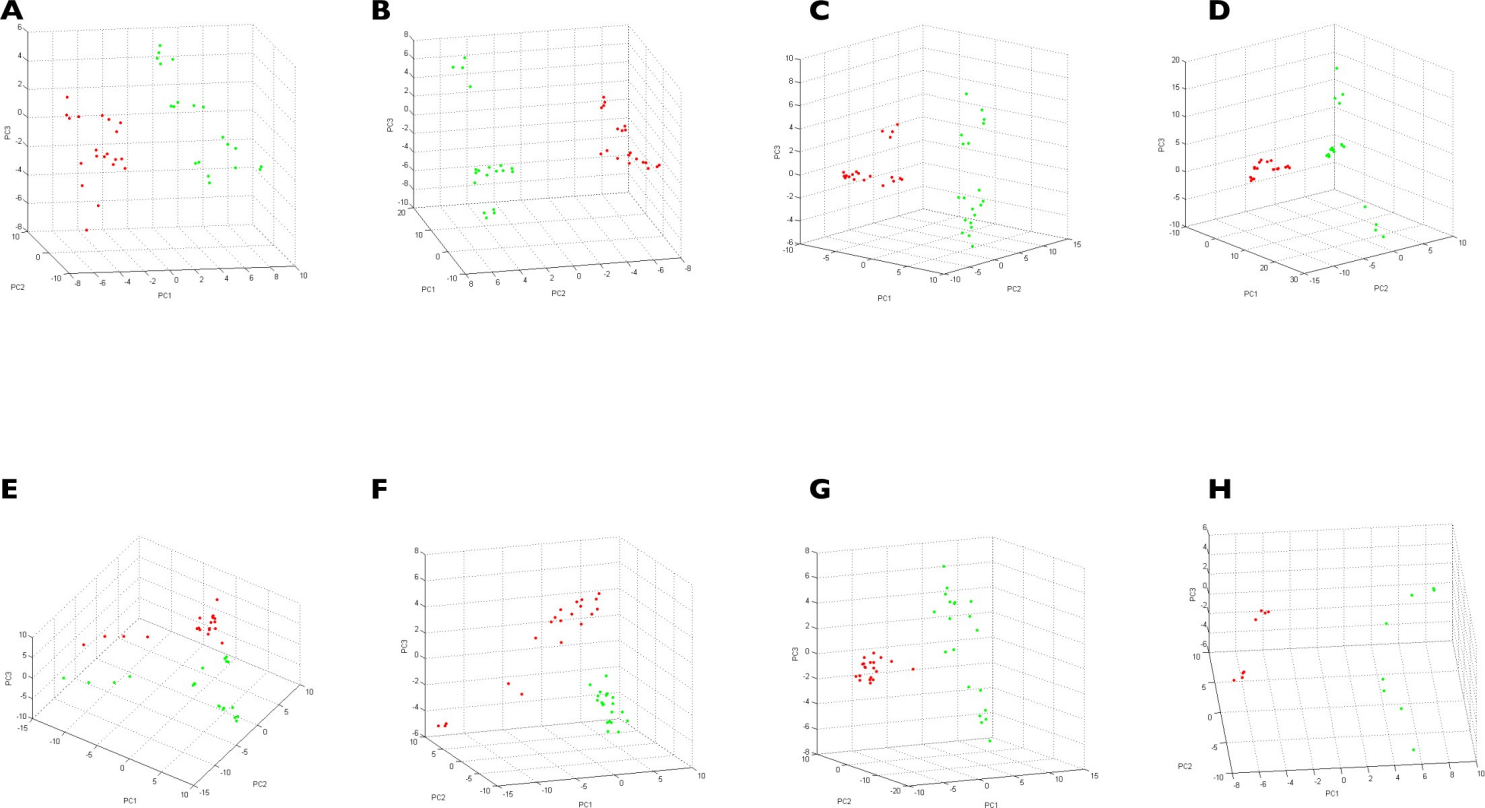
Assessment of MS spectra specificity according to species and body parts using principal component analysis. MS spectra from the legs and half–idiosoma of ticks were analyzed by species using the PCA tool. Red dots represent spectra of the four legs and green dots represent spectra of the half–idiosoma. (A) *D*. *reticulatus* (B) *I*. *acuminatus* (C) *I*. *persulcatus* (D) *I*. *ricinus* (E) *I*. *scapularis* (F) *I*. *uriae* (G) *I*. *ventalloi* (H) *I*. *vespertilionis*.

To confirm reproducibility and specificity of MS spectra according to body parts by species, PCAs were performed (Fig 4). PCAs revealed clustering in two groups of the dots corresponding to MS spectra from legs and half-idiosoma. This finding supports the specificity of MS profiles between these two body parts for each of the eight species tested. Collectively, these results yielded unique reproducible MS spectra for each tick species tested according to body parts.

CCI matrix also revealed the correlation of MS spectra between specimens of the same species per body parts (0.57 ± 0.23 for legs: 0.57 ± 0.24 for half-idiosoma; S2 Fig). Conversely, lower CCI were obtained between *Ixodes* species and body parts (0. 13 ± 0.08 for legs and 0.14 ± 0.07 for half-idiosoma; S2 Fig) confirming the reproducibility and specificity of protein profiles according to tick species and body part.

#### Assignment of discriminating peaks

To identify discriminatory peaks among the nine *Ixodes* tick species for each body part, the Genetic Algorithm (GA) tool from ClinProTools^™^ software was used. The GA model exhibited a pattern of 67 and 88 discriminatory mass peaks between *Ixodes* tick species for legs and half-idiosoma, respectively (S3 & S4 Tables and S1 Fig). The presence or absence of these discriminatory peaks per tick species displayed RC and CV values of 100% and 95.8%, respectively, for MS spectra from legs. For MS spectra from half-idiosoma, RC and CV values of 100% and 99.5% were obtained, respectively (Table 3).

**Table 3:** Performance of the genetic algorithm based on the presence or absence of discriminatory peaks of the *Ixodes* species. The analysis was not performed for *I*. *frontalis* and *I*. *hexagonus* for the half–idiosoma because all specimens were engorged.

	4 legs		Half-idiosoma	
Species	Recognition capability	Cross validation	Recognition capability	Cross validation
*I*. *frontalis*	100%	100%		
*I*. *hexagonus*	100%	89.66%		
*I*. *acuminatus*	100%	97.62%	100%	100%
*I*.* persulcatus*	100%	96.55%	100%	96.67%
*I*.* ricinus*	100%	90.7%	100%	100%
*I*.* scapularis*	100%	90%	100%	100%
*I*. *uriae*	100%	100%	100%	100%
*I*. *ventalloi*	100%	97.5%	100%	100%
*I*. *vespertilionis*	100%	100%	100%	100%
**Total**	**100%**	**95.78%**	**100%**	**99.52%**

### Blind tests

Accuracy of tick identification by MALDI-TOF MS was tested using 202 morphologically identified specimens representing the 10 tick species included in the MS reference database. The query of the MS database with MS profiles for legs showed that 96.5% of the specimens (n = 195/202) obtained an LSV of 1.8 or higher corresponding to the threshold defined for relevant identification; thus, confirming the morphological classification (S1 Table). The four-leg MS analysis confirmed the morphological identification for six of the seven specimens which did not reach the LSV threshold.

The 46 engorged ticks were excluded from half-idiosoma MS analysis. The rate of relevant (LSV >1.8) identifications using half-idiosoma MS spectra queried against the MS database was ˃91.0% (n = 142/156). Concordance of tick species identification between morphological and MS analyses was obtained for all half-idiosoma MS spectra queried against the MS database reaching the LSV threshold (S1 Table and Table 4).

**Table 4:** Results of the blind test procedure against the four legs and half–idiosoma database.

Species	No. of specimens used for the blind test	LSVs* [Low-High]	Top species identified^$^	Differences in LSVs between the first and second top species [Mean ± SD]^§^
*4 legs*				
*I*.* ventalloi*	12	[1.92–2.30] (11)	*I*.* ventalloi*	0.81±0.21
		*[1*.*42] (1)*	/	
*I*.* ricinus*	104	[1.8–2.41] (99)	*I*.* ricinus*	0.73±0.15
		*[1*.*57–1*.*78] (5)*	/	
*I*.* persulcatus*	13	[2.02–2.43] (13)	*I*.* persulcatus*	0.97±0.15
*I*.* scapularis*	22	[1.80–2.84] (22)	*I*.* scapularis*	0.94±0.29
*I*.* acuminatus*	8	[2.24–2.65] (8)	*I*.* acuminatus*	1.15±0.19
*I*.* uriae*	8	[1.98–2.46] (7)	*I*.* uriae*	2.28±0.17
		*[1*.*74 ] (1)*	/	
*I*.* vespertilionis*	2	[2.19–2.45] (2)	*I*.* vespertilionis*	1.33±0.22
*I*.* hexagonus*	23	[1.87–2.54] (23)	*I*.* hexagonus*	1.14±0.35
*I*.* frontalis*	2	[2.15–2.43] (2)	*I*.* frontalis*	1.14±0.29
*D*.* reticulatus*	8	[2.23–2.51] (8)	*D*.* reticulatus*	1.42±0.16
*Half-idiosoma*				
*I*.* ventalloi*	5	[1.81–2.13] (5)	*I*.* ventalloi*	0.22±0.31
*I*.* ricinus*	90	[1.92–2.64] (87)	*I*.* ricinus*	0.51±0.19
		*[0*.*58–1*.*59] (3)*	/	
*I*.* persulcatus*	13	[1.81–2.41] (11)	*I*.* persulcatus*	0.67±0.39
		*[1*.*62–1*.*68] (2)*	/	
*I*.* scapularis*	22	[1.81–2.29] (15)	*I*.* scapularis*	0.43±0.17
		*[1*.*44–1*.*77] (7)*	/	
*I*.* acuminatus*	8	[2.25–2.67] (8)	*I*.* acuminatus*	0.82±0.41
*I*.* uriae*	8	[1.90–2.21] (6)	*I*.* uriae*	1.28±0.14
		*[1*.*46–1*.*65] (2)*	/	
*I*.* vespertilionis*	2	[2.06–2.08] (2)	*I*.* vespertilionis*	1.44±0.21
*D*.* reticulatus*	8	[2.11–2.53] (8)	*D*.* reticulatus*	1.53±0.41

Incorrect identifications are shown in italics

*Range of log score values (LSVs), the number of specimens included in each range of LSVs (above and below 1.8) are indicated into brackets

^$^Names of the first top hit species identified with relevant LSVs (LSVs >1.8)

^§^Mean and standard deviation of the differences in log score values between the first and second top species identified by MS

To assess the risk of tick species misidentification using MALDI-TOF MS, the differences in LSVs (dLSVs) between the first and second top distinct species identified for each body part were calculated (S1 Table and Table 4). The dLSVs of *Ixodes* ticks ranged from 0.73 to 1.41 for legs and from 0.22 to 1.44 for half-idiosoma. As these values were expressed in logarithmic scale, these differences could be considered substantial. Moreover, the query of legs and half-idiosoma against the commercial bacteria MS spectra database revealed no cross-identification. This confirms the specificity of the MS profiles. All LSVs were lower than the significant threshold of 1.8.

**Fig 5:**
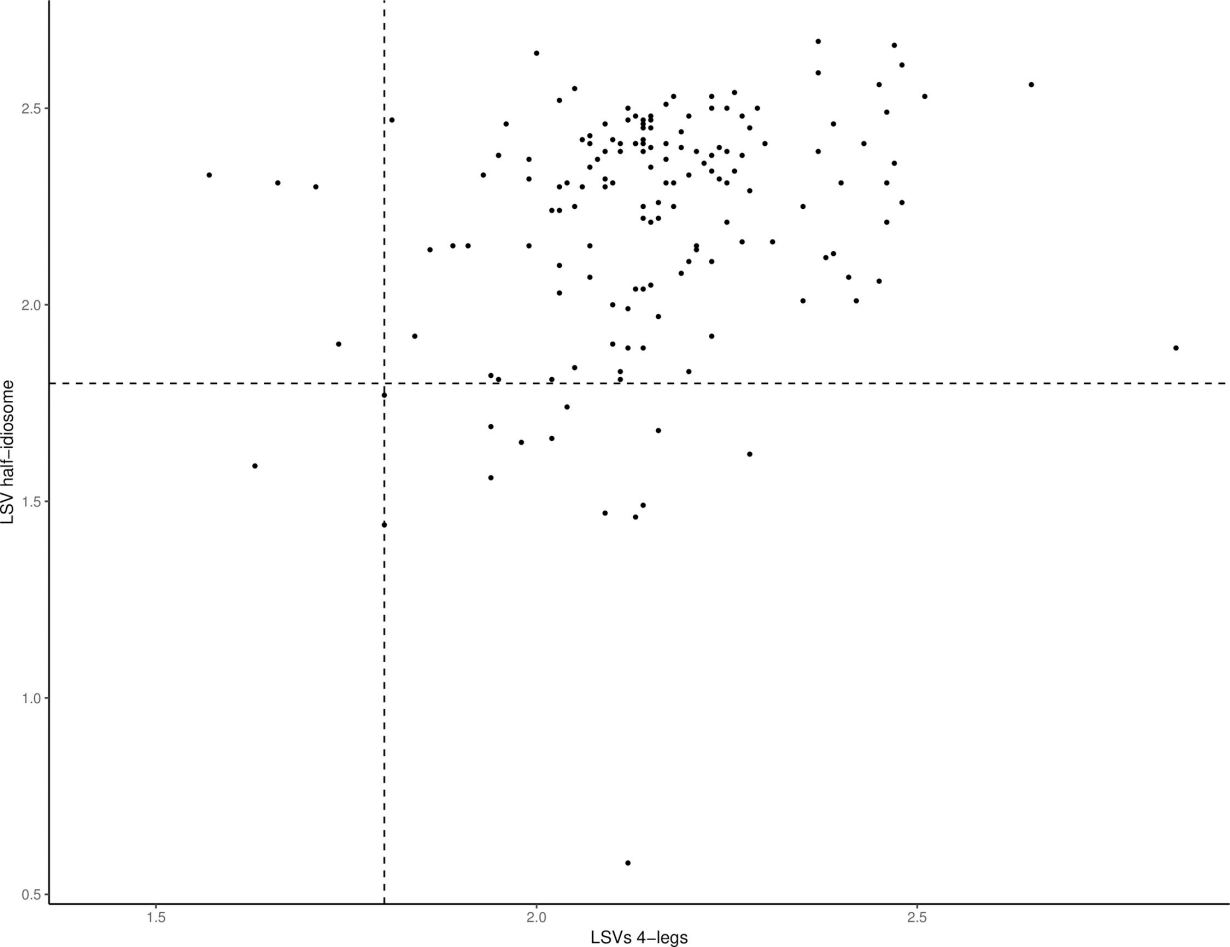
Comparison of LSVs from MS spectra of the 10 tick species according to body part. Specimens, for which the four legs and half–idiosoma were available, are presented (n = 156). The dashed line represents the threshold value for relevant identification (LSVs >1.8). LSV, log score value.

Interestingly, the combination of MS identification results from legs and half-idiosoma increased the rate of relevant identifications to 99.4% (n = 155/156) for specimens for which both body parts were submitted to MS analysis (Fig 5). Species identification for each body part was 100% concordant and corroborates morphological identification. The only tick identification considered unreliable (LSVs of 1.63 and 1.59 for legs and half-idiosoma, respectively) was classified as *I*. *ricinus* according to both body parts; thus confirming the morphological result. Finally, among the 202 tick specimens submitted to MS analysis for identification, only three failed to reach the LSV threshold value for relevant identification, with at least one of the body parts. The legs or both body parts validated the morphological identification for two of them, and the remaining specimen morphologically classified as *I*. *ventalloi* was classified by leg MS spectra as *I*. *ricinus*. The low MS identification score (LSV = 1.42) reflected the poor quality of the respective MS spectra with few MS peaks of low intensity (<2,000 arbitrary units). The global proportion of relevant identifications was 98.5% (n = 199/202). This rate can be considered very interesting as nine tick species belonged to the same *Ixodes* genus, including several species with very close morphological features, especially at the nymphal stages.

## Discussion

MALDI-TOF MS profiling emerged a decade ago as an innovative strategy for rapid, low-cost identification of arthropods, especially for vectors of infectious diseases [9,17,34,35]. Contrary to genome which is identical for all body parts of specimens, MS spectra are different according to body parts (legs, capitulum, idiosoma) [36], and other factors can modify them such as the blood-engorged status [17], or infectious status [37–39]. To facilitate comparisons and to share MS spectra database, guidelines for sample preparation, storing modes, or body part selection were developed for some arthropod families [19,20]. MALDI-TOF MS technology has so far been successfully used for identifying fresh [17] or alcohol-preserved tick specimens [18,40] using ticks’ legs as biological material. However, databases built for these studies have only included a few tick species belonging to the same genus. Effective assessment of this innovative tool’s effectiveness in correctly classifying closely related tick species was thus difficult.

In order to improve the rate and accuracy of MS identification, two distinct body parts (legs and half-idiosoma of ticks) were submitted to MS analysis. A previous study comparing MS profiles from various *I*. *ricinus* body parts reported distinct protein patterns between legs and half-idiosoma from this species of ticks [21]. The present study thus assessed whether half-idiosoma could also be used as a relevant body part for tick species identification using a MALDI-TOF MS profiling strategy and whether it could improve identification accuracy. Legs and half-idiosoma of ticks are therefore proposed to be systematically submitted to MS analysis to improve reliability of tick species identification.

Reproducible interspecies-specific MS spectra were obtained for each tick species. Although variations occurred according to sex type or developmental stages, no clear distinction was observed on the MSP dendrograms among specimens of the same species according to sex type (male vs. female) or developmental stages (adults vs. nymphs). These results reveal the lower impact of these factors on MS profiles and suggest that the main determinant of MS profiles is the species. Karger et al. [17] previously observed this phenomenon by comparing MS spectra from ticks at various developmental stages or sex type using whole specimens.

The comparison of dendrograms from legs and from half-idiosoma showed that the topology of the trees is different despite the use of paired body parts. The present results confirmed that half-idiosoma have similar characteristics as legs, and are relevant for tick species identification by MS. Distinct topologies of the MSP dendrograms between paired legs and half-idiosoma from tick specimens also suggested that each body part generates distinct reproducible MS spectra. PCA analyses confirmed the singularity of MS profiles for each body part from each tick species. The reproducibility and specificity of protein profiles per tick species and per body part were objectified by CCI, suggesting that each body part can be tested in an independent manner. Reference MS database query with protein profiles from legs and half-idiosoma from the same tick specimen thus constitutes a double-independent species identification. This double tick species identification checking is frequently performed by sequencing two or more distinct gene targets using molecular tools, as performed in the present study to improve the accuracy of molecular identification [7].

Among the specimens included in the present study which both body parts were submitted to MS analysis, the rates of relevant (LSVs >1.8) identifications were 96.8% and 91.0% for legs and half-idiosoma, respectively. Combining results from both body parts increased the rate of relevant identifications to 99.4% with 100% of corroborative tick species classification concordant with morphological identification. Among the remaining 46 specimens for which only the legs were submitted to MS analysis, 44 were properly identified. Finally, only three specimens were not reliably identified, two *I*. *ricinus* nymphs and one *I*. *ventalloi* female. The global identification rate was 98.5% (199/202).

Interestingly, a lower rate of relevant identifications was obtained for half-idiosoma than for legs. The presence of residues in the midgut, such as residual blood meal which can persist for several weeks or months following the blood meal [41] could explain the minor changes in MS profiles; thus decreasing the scores of spectra matching with MS DB. Karger and collaborators [22] recently reported spectral variations between *I*. *ricinus* specimens according to geographical origin, environmental factors, and seasons. These slight MS profile changes only concern the intensity of a few peaks, which does not prevent specimen identification [17]. Moreover, ticks presented in this study − especially *I*. *ricinus* ticks − were collected at different times and in various geographical regions, and 96.7% (87/96) of *I*. *ricinus* specimens were correctly identified using half-idiosoma. The three specimens that were not identified had spectra of low quality, explaining their low LSVs.

Using four legs for tick identification by MALDI-TOF MS analysis has several advantages. The whole body is preserved allowing additional morphological and/or molecular analyses (*e*.*g*., taxonomy validation or pathogen search). Leg MS spectra remain unchanged irrespective of the engorgement status of the tick [27]. Conversely, recent blood meals can compromise tick species identification using half-idiosoma [17]. Indeed, for ticks collected from hosts (*i*.*e*., humans or animals), confirmation of tick species using half-idiosoma cannot always be performed. It depends on the engorgement status, and thus limits its diagnostic use. Nevertheless, the inability to reach LSV threshold for relevant tick identification only using leg MS spectra has been repeatedly reported [18,27,42]. These questionable identifications were usually attributed to the poor MS spectra “quality” (decreased peak intensity and diversity). This problem was particularly observed with specimens at immature stages (*i*.*e*., larval and nymphal stages) [17]. The small size of specimens at these immature stages is associated with fastidious dissection of legs with low quantity of extracted proteins as previously reported for early stages of mosquito larvae [43]. Intensities of four-leg MS spectra were indeed sometimes low, whereas intensities of half-idiosoma MS spectra were higher. This difference is probably due to the larger amount of proteins extracted from half-idiosoma.

As immature stages are usually preponderant during field collection of ticks [44,45], confirmation of tick species identity using a second body part at the same time or with two-tiered testing could rule out equivocal classifications. More than half of the ticks submitted to MS analysis in the present study were at the nymphal stage. As questing ticks are usually not engorged, half-idiosoma could be a helpful additional strategy.

The present work included nine species of the *Ixodes* genus, among which three (*I*. *ricinus*, *I*. *scapularis*, and *I*. *persulcatus*) are members of the *I*. *ricinus* complex [23]. For bacterial identification, closely related species are difficult to identify with MALDI-TOF MS. For example, *Streptococcus pseudopneumoniae* and *S*. *pneumoniae* are hard to differentiate routinely [46] as well as *Escherichia coli* and *Shigella* spp [47]. To our knowledge, there is no previous study assessing MALDI-TOF ability to discriminate and identify the closely related tick species of the *I*. *ricinus* complex. Indeed, no misidentification between *I*. *ricinus*, *I*. *persulcatus* and *I*. *scapularis* had been noticed during this work. Moreover, MALDI-TOF allows for the correct identification subadult specimens (*i*.*e*., nymphs or larvae) for which morphological identification is harder than for adult specimens even for well-trained taxonomists and are, for the study of Lyme borreliosis, the main material collected [11,48].

Correct identification of tick species is the crucial first step for all tick-associated researches. Assessing the risk of tick-borne pathogen, vector distribution, and vector/host associations can indeed be distorted when misidentifications occur [49]. MALDI-TOF MS technology is a suitable method for high throughput species identification of field-collected specimens. Furthermore, some of the ticks included in this database − *I*. *ricinus*, *I*. *persulcatus*, *I*. *hexagonus*, *I*. *scapularis*, *I*. *uriae* − are vectors of *B*. *burgdorferi* sl, the causative agent of Lyme disease, the most prevalent tick borne disease in the Northern hemisphere [5]. *I*. *ricinus*, *I*. *persulcatus*, and *I*. *scapularis* are also vectors of *Anaplasma phagocytophilum*, responsible for human granulocytic anaplasmosis [50]. *Borrelia miyamotoi* [51] and tick-borne encephalitis virus [52] can be transmitted by *I*. *ricinus* and *I*. *persulcatus*, respectively. *Dermacentor reticulatus* is a vector of *Rickettsia slovaca* and *R*. *raoultii*, responsible for tick-borne lymphadenopathy (TIBOLA) [53]. Other ticks included in this database such as *I*. *frontalis* do not seem to be competent for the transmission of *B*. *burgdorferi* sl. [6], although potentially playing a role in transmitting the bacterium by co-feeding. Hence, accurate identification of ticks removed from hosts or collected in the field allows to distinguish tick vectors of TBDs from non-vectors and helps to orientate pathogen diagnosis and control strategies.

The present study also identified a limitation related to the genomic database. No sequence of *I*. *acuminatus* was available on the GenBank database. The 2% difference between the COI sequence of *I*. *acuminatus* and *I*. *redikorzevi* COI can be explained by *I*. *redikorzevi* being a synonym for *I*. *acuminatus*, as suggested by several authors [54]. Sequences may correspond to intraspecific variability. It should be noted that the GenBank sequence of *I*. *redikorzevi* form a monophyletic group with our *I*. *acuminatus* sequences (Fig 1B).

Interestingly, interrogating the GenBank database with 16s rDNA from the specimens identified as *I*. *acuminatus* revealed 100% similarity with a sequence of *I*. *ricinus* (Accession number JN248424.2). This *I*. *ricinus* sequence is different from the remaining sequence of *I*. *ricinus*, either already available from GenBank (KF197115.1, NC 002010.1) or from our own sequences of *I*. *ricinus*. This JN248424.2 sequence of the *I*. *ricinus* mitogenome [55] harbors regions of low sequence identity with the 18 mitogenomes obtained by Carpi et al. [56], leading those authors to exclude the sequence of Montagna et al. [55] from their analysis.

As previously reported, MALDI-TOF MS does not seem to be an appropriate method for phylogenetic studies [17,27]. Trees built based on the data provided by MALDI-TOF MS cannot be analyzed using phylogenetical methods, the clustering method can thus only be a phenetic method, clustering samples according to their overall similarities. Moreover, all phylogenetical methods are based on the comparison of homologous sites. With MALDI-TOF MS data, the analyzed peaks may not be all homologous.

All tick-associated researches are based on the correct initial identification of tick species. Assessing the risk of tick-borne pathogen, vector distribution, and vector/host associations can indeed be distorted when misidentifications occur [49]. MALDI-TOF MS technology is a suitable method for high throughput species identification of field-collected specimens. Furthermore, some of the ticks included in this database − *I*. *ricinus*, *I*. *persulcatus*, *I*. *hexagonus*, *I*. *scapularis*, *I*. *uriae* − are vectors of *Borrelia burgdorferi* sl, the causative agent of Lyme disease [5]. *I*. *ricinus*, *I*. *persulcatus*, and *I*. *scapularis* are also vectors of *Anaplasma phagocytophilum*, responsible for human granulocytic anaplasmosis [50]. *Borrelia miyamotoi* [51] and tick-borne encephalitis virus [52] can be transmitted by *I*. *ricinus* and *I*. *persulcatus*, respectively. *Dermacentor reticulatus* is a vector of *Rickettsia slovaca* and *R*. *raoultii*, responsible for tick-borne lymphadenopathy (TIBOLA) [53]. Other ticks included in this database such as *I*. *frontalis* do not seem to be competent for the transmission of *B*. *burgdorferi* sl. [6], although potentially playing a role in transmitting the bacterium by co-feeding. Hence, accurate identification of ticks removed from hosts or collected in the field allows to distinguish tick vectors of TBDs from non-vectors and helps to orientate pathogen diagnosis and control strategies.

## Conclusion

The present study demonstrated that for tick identification both legs and half-idiosoma can be used as a matrix for MALDI-TOF MS identification. In this study, this high throughput tool has been employed for the identification of closely related species belonging to the *Ixodes* genus which are hardly distinguishable using morphological tools. MALDI-TOF MS thus discriminates between tick vectors of Lyme disease and non-vectors which is of utmost importance for large scale epidemiological studies and “live” monitoring of field-collected tick vectors.

The double-check strategy proposed herein, based on the combined use of two matrices (half-idiosoma and tick legs) improves the accuracy of this method. The database set-up constitutes the foundation stone for a larger and shared database.

## Supporting information

S1 Fig Discriminatory peaks between the *Ixodes* species for legs (A) and half-idiosoma (B).
(PDF)

S2 Fig Evaluation of *Ixodes* MS spectra reproducibility and specificity according to tick species and body parts using composite correlation index (CCI). MS spectra from two to five specimens per body part were analyzed using the CCI tool. Tick species and body part are indicated on the left side of the heat map. Levels of MS spectra reproducibility are indicated in red and blue revealing relatedness and incongruence between spectra, respectively. CCI matrix was calculated using MALDI-Biotyper v3.0. software with default settings (mass range 3.0 ± 12.0 kDa; resolution 4; 8 intervals; auto-correction off). The values correspond to the mean coefficient of correlation and respective standard deviations obtained for paired condition comparisons.
(PDF)

S1 Table Detailed origins and results of the blind test procedure of the ticks’ body parts. (XLSX)

S2 Table Sequences obtained in this study and submitted in the GeneBank database. (XLSX)

S3 Table Mass peak list distinguishing *Ixodes* tick species using legs as matrix, determined by Genetic Algorithm model analysis of ClinProTools. (DOCX)

S4 Table Mass peak list distinguishing *Ixodes* tick species using half-idiosoma as matrix, determined by Genetic Algorithm model analysis of ClinProTools. (DOCX)

S1 File Raw MS spectra from legs and half-idiosoma of ticks are included in the MS reference database. MS spectra were obtained using Microflex LT MALDI-TOF Mass Spectrometer (Bruker Daltonics).
(ZIP)
