# Supplementary figures and images for: Identification of closely related Ixodes species by protein profiling with MALDI-TOF mass spectrometry
Source: PLoS One. 2019 Oct 17;14(10):e0223735. doi: 10.1371/journal.pone.0223735 (PMC6797106; doi:10.1371/journal.pone.0223735)

(A)

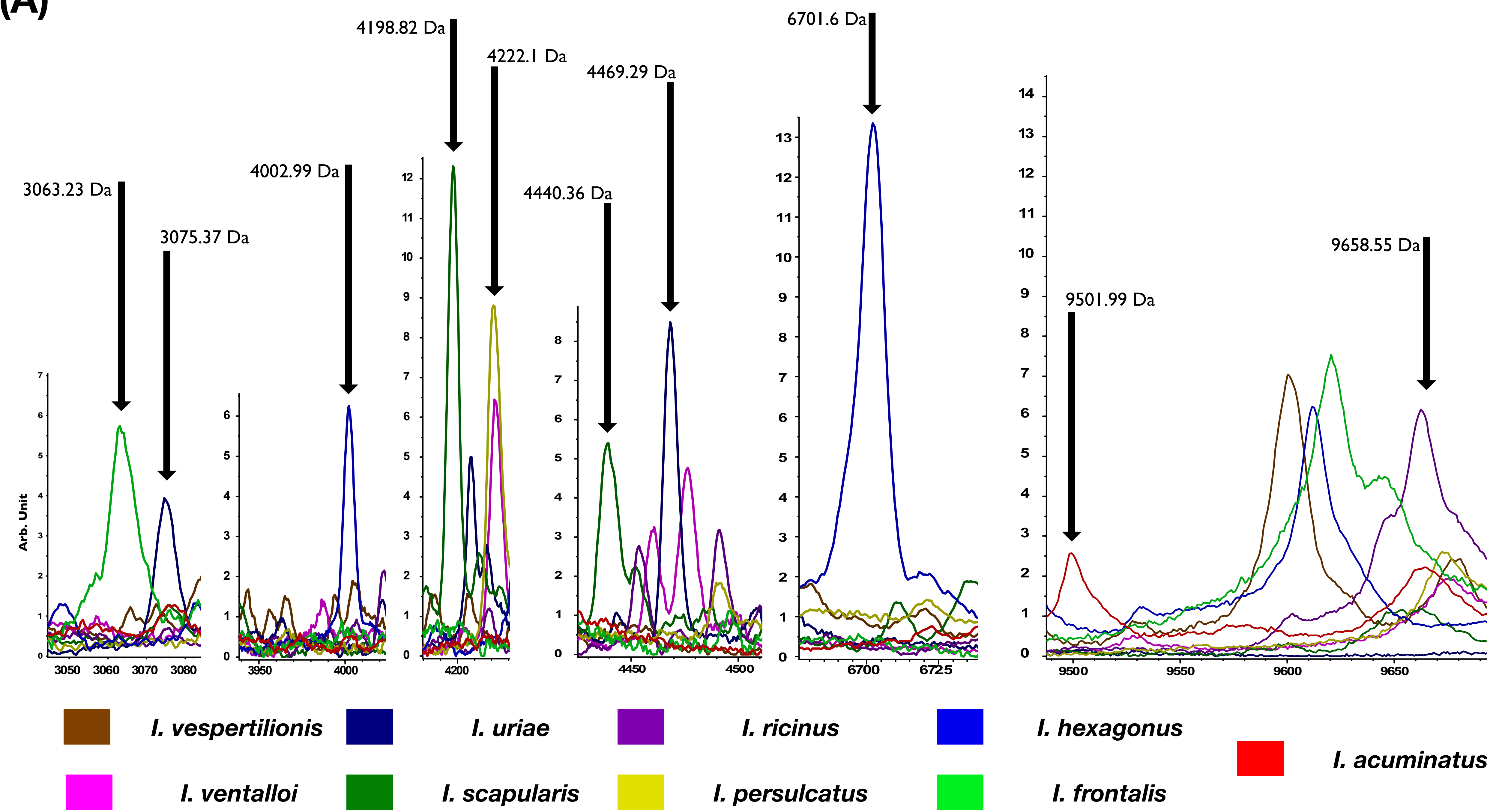

(B)

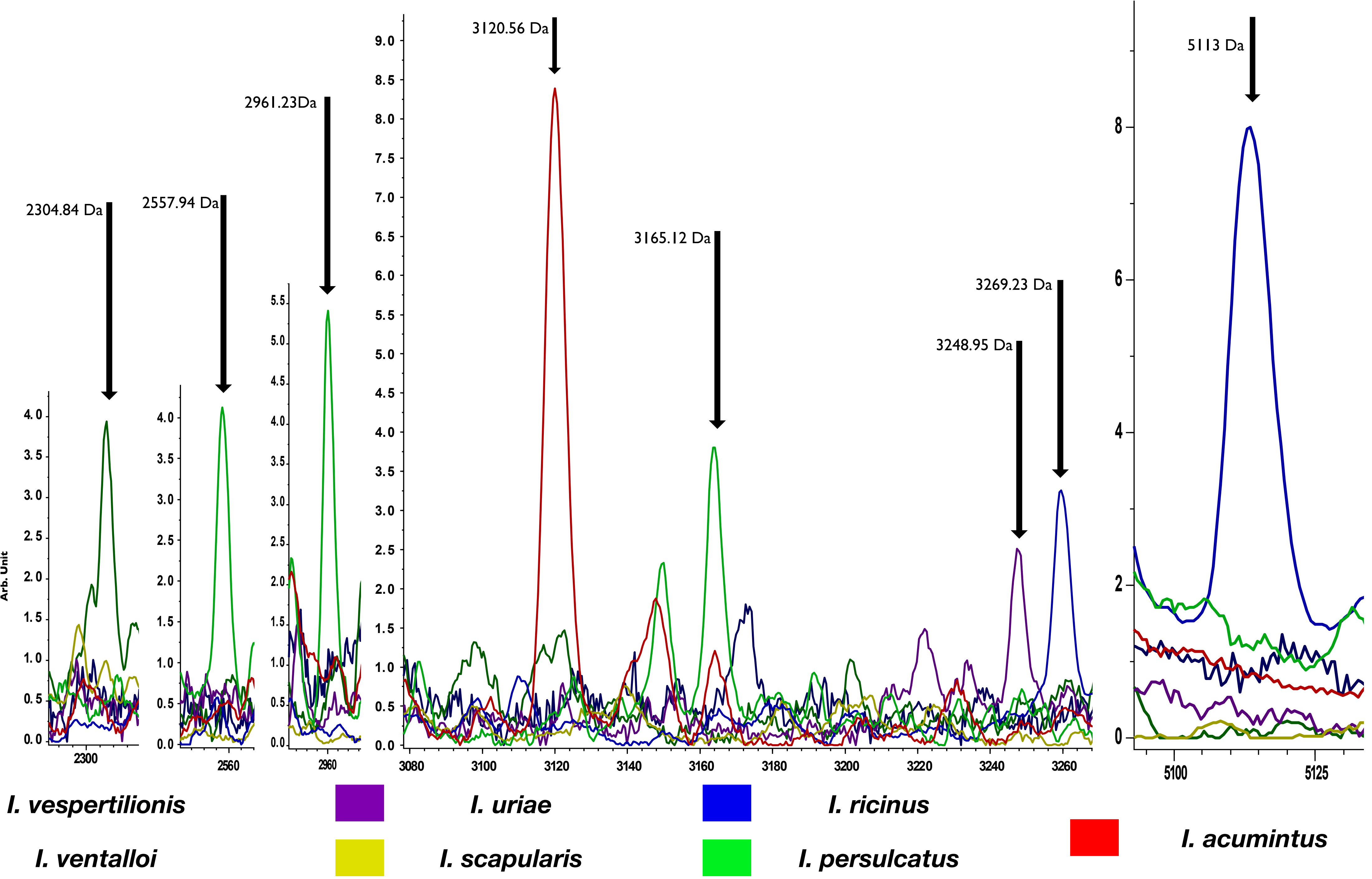

Supplement: S1 Fig — Discriminatory peaks between the Ixodes species for legs (A) and half-idiosoma (B). (PDF) [file pone.0223735.s001.pdf]

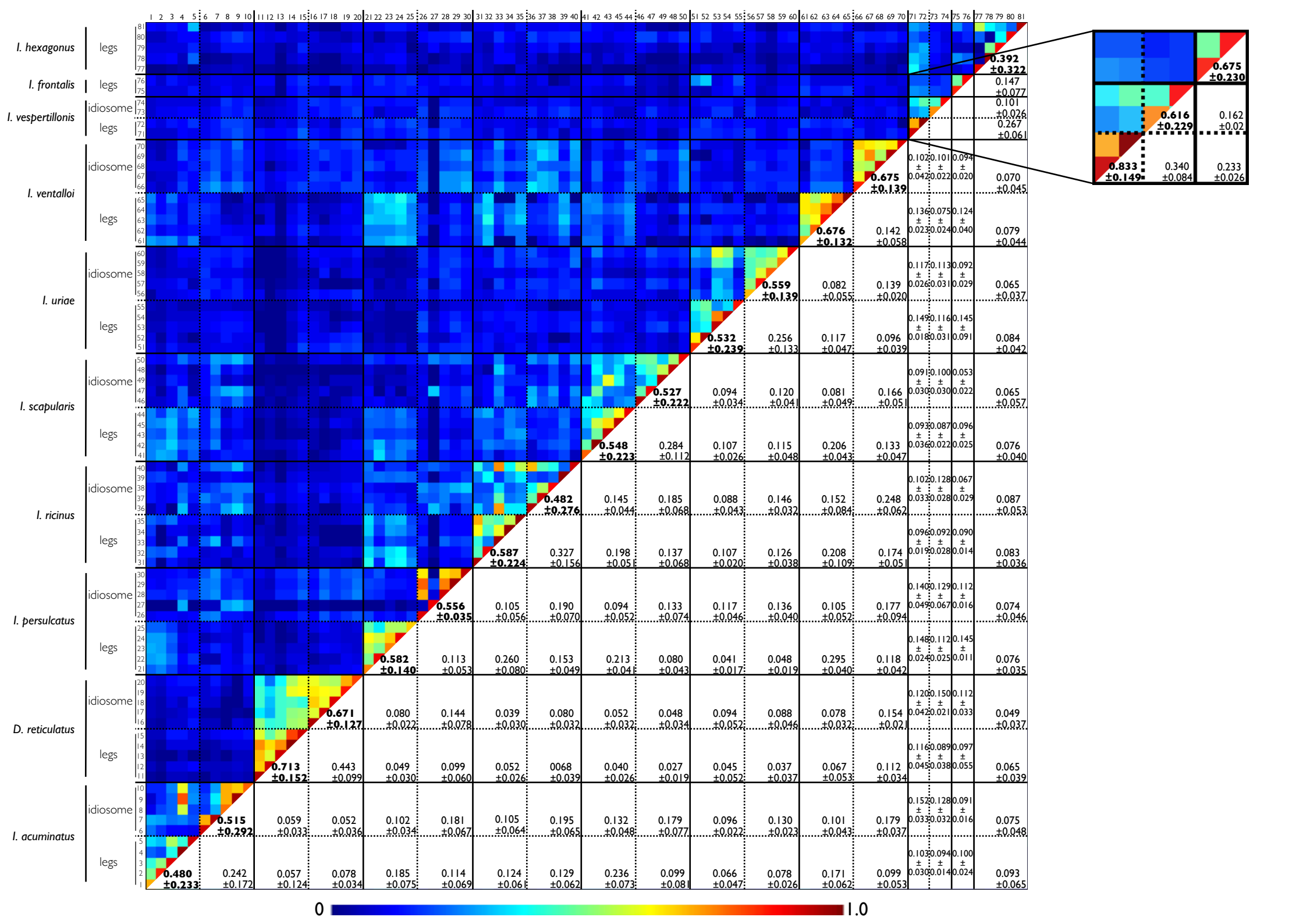

Supplement: S2 Fig — MS spectra from two to five specimens per body part were analyzed using the CCI tool. Tick species and body part are indicated on the left side of the heat map. Levels of MS spectra reproducibility are indicated in red and blue revealing relatedness and incongruence between spectra, respectively. CCI matrix was calculated using MALDI-Biotyper v3.0. software with default settings (mass range 3.0 ± 12.0 kDa; resolution 4; 8 intervals; auto-correction off). The values correspond to the mean coefficient of correlation and respective standard deviations obtained for paired condition comparisons. (PDF) [file pone.0223735.s002.pdf]
