# Supplementary material for: Identification of closely related Ixodes species by protein profiling with MALDI-TOF mass spectrometry
Source: PLoS One. 2019 Oct 17;14(10):e0223735. doi: 10.1371/journal.pone.0223735 (PMC6797106; doi:10.1371/journal.pone.0223735)
Supplement: S3 Table — (DOCX) [file pone.0223735.s005.docx]

**S3 Table.** Mass peak list distinguishing *Ixodes* tick species using legs as biologic material, based on the Genetic Algorithm model analysis of ClinProTools.

| **Mass m/z [Da]*** | ***I. acuminatus*** | ***I. vespertillionis*** | ***I. ricinus*** | ***I. persulcatus*** | ***I. ventalloi*** | ***I. uriae*** | ***I. scapularis*** | ***I. frontalis*** | ***I. hexagonus*** |
| --- | --- | --- | --- | --- | --- | --- | --- | --- | --- |
| 2030.71 | - | - | - | - | **+** | - | - | - | - |
| 2146.99 | - | - | - | - | **+** | - | - | - | - |
| 2170.05 | - | - | - | - | - | - | - | **+** | - |
| 2273.33 | - | - | - | - | - | - | - | **+** | - |
| 2289.31 | - | - | - | - | - | - | - | **+** | **+** |
| 2318.23 | - | - | - | - | **+** | - | - | - | - |
| 2427.81 | - | - | - | - | **+** | - | - | - | - |
| 2465.51 | - | - | - | - | - | - | - | - | **+** |
| 2489.76 | - | - | - | - | - | - | - | - | **+** |
| 2565.69 | - | - | - | - | - | - | - | - | **+** |
| 2600.08 | - | - | - | **+** | - | - | - | - | **+** |
| 2947.66 | **+** | - | - | - | - | - | - | - | - |
| 3063.23 | - | - | - | - | - | - | - | **+** | - |
| 3075.37 | - | - | - | - | - | **+** | - | - | - |
| 3406.1 | - | - | - | - | - | - | **+** | - | - |
| 3461.71 | - | - | - | - | - | - | - | - | **+** |
| 3495.92 | - | - | - | - | - | - | **+** | - | - |
| 3732.38 | - | - | **+** | - | - | - | - | - | - |
| 3831.27 | - | - | - | - | - | - | - | - | **+** |
| 4002.99 | - | - | - | - | - | - | - | - | **+** |
| 4198.82 | - | - | - | **+** | - | - | **+** | - | - |
| 4222.1 | **+** | - | **+** | - | - | - | - | - | - |
| 4274.3 | **+** | - | **+** | - | - | - | - | - | - |
| 4347.34 | - | - | - | - | - | - | - | **+** | **+** |
| 4413.58 | **+** | - | - | - | - | - | - | - | - |
| 4440.36 | - | - | - | - | - | - | **+** | - | - |
| 4469.29 | - | - | - | - | - | **+** | - | - | - |
| 4652.73 | - | - | **+** | - | - | - | - | **+** | **+** |
| 4696.95 | - | - | - | - | - | - | - | **+** | - |
| 4758.61 | - | - | - | - | - | - | - | - | **+** |
| 4807.75 | - | - | - | - | - | - | - | **+** | **+** |
| 4860.64 | - | - | - | - | - | - | **+** | **+** | - |
| 5061.37 | - | - | - | - | - | - | - | - | **+** |
| 5133.12 | - | - | - | - | - | - | - | - | **+** |
| 5198.45 | - | - | - | - | - | - | - | - | **+** |
| 5433.5 | - | - | - | - | - | - | - | - | **+** |
| 5875.14 | - | - | - | - | - | **+** | - | - | - |
| 6315.61 | - | - | - | - | - | **+** | - | - | - |
| 6384.18 | - | - | - | - | - | **+** | - | - | - |
| 6701.6 | - | - | - | - | - | - | - | - | **+** |
| 6804.63 | - | - | - | - | - | - | - | - | **+** |
| 7179.44 | - | - | - | - | - | - | - | - | **+** |
| 7869.61 | - | **+** | - | - | - | - | - | - | - |
| 9078.5 | - | - | - | - | - | **+** | - | - | - |
| 9501.39 | **+** | - | - | - | - | - | - | - | - |
| 9658.55 | - | - | - | - | **+** | - | - | - | - |
| 9867.48 | - | - | - | - | - | **+** | - | - | - |
| 9988.7 | **+** | - | - | - | - | - | - | - | - |
| 10406.62 | - | - | - | - | - | - | - | - | **+** |
| 11193.35 | - | - | - | - | **+** | - | - | - | - |
| 11235.23 | - | **+** | - | - | - | - | - | - | - |
| 11490.49 | - | **+** | - | - | - | - | - | - | - |
| 11740.51 | - | **+** | - | - | - | - | - | - | - |
| 12357.37 | - | - | - | - | **+** | - | - | - | - |
| 12625.13 | - | **+** | - | - | - | - | - | - | - |
| 12759.77 | - | **+** | - | - | - | - | - | - | - |
| 13066.34 | - | - | - | - | **+** | - | - | - | - |
| 13236.04 | - | - | - | - | **+** | - | - | - | - |
| 15593.63 | - | **+** | - | - | - | - | - | - | - |
| **Total** | **6** | **7** | **4** | **2** | **9** | **6** | **5** | **9** | **19** |

*List included uniquely species specific mass peaks. Da, Daltons; m/z, mass to charge.
