# Supplementary material for: Identification of closely related Ixodes species by protein profiling with MALDI-TOF mass spectrometry
Source: PLoS One. 2019 Oct 17;14(10):e0223735. doi: 10.1371/journal.pone.0223735 (PMC6797106; doi:10.1371/journal.pone.0223735)
Supplement: S4 Table — (DOCX) [file pone.0223735.s006.docx]

**S4 Table.** Mass peak list distinguishing *Ixodes* tick species using half-idiosoma as biologic material, based on the Genetic Algorithm model analysis of ClinProTools.

| **Mass m/z [Da]*** | ***I. acuminatus*** | ***I. vespertillionis*** | ***I. ricinus*** | ***I. persulcatus*** | ***I. ventalloi*** | ***I. uriae*** | ***I. scapularis*** |
| --- | --- | --- | --- | --- | --- | --- | --- |
| 2304.84 | - | - | - | - | **+** | - | - |
| 2368.56 | **+** | **+** | **+** | - | - | - | - |
| 2386.91 | - | - | - | - | - | **+** | - |
| 2425.79 | - | - | - | **+** | - | - | **+** |
| 2557.94 | - | - | - | **+** | - | - | - |
| 2721.77 | - | **+** | - | - | - | - | - |
| 2788.58 | - | - | - | **+** | - | - | - |
| 2804.61 | - | - | - | **+** | - | - | - |
| 2817.62 | - | - | - | - | **+** | - | - |
| 2840.22 | - | - | - | - | **+** | - | - |
| 2862.77 | **+** | - | - | - | - | - | - |
| 2904.33 | **+** | - | - | - | - | - | - |
| 2961.23 | - | - | - | **+** | - | - | - |
| 2984.1 | - | **+** | - | - | - | - | - |
| 3120.56 | **+** | - | - | - | - | - | - |
| 3165.12 | - | - | - | **+** | - | - | - |
| 3248.95 | - | - | - | - | - | **+** | - |
| 3260.23 | - | - | **+** | - | - | - | - |
| 3329.73 | - | - | - | **+** | - | - | - |
| 3354.78 | - | - | - | - | - | - | **+** |
| 3404.07 | - | - | - | - | - | - | **+** |
| 3498.65 | - | - | - | - | - | - | **+** |
| 3520.57 | - | - | - | - | - | - | **+** |
| 3659.21 | **+** | - | - | - | - | - | - |
| 3686.24 | - | - | - | - | - | - | **+** |
| 3725.86 | - | **+** | - | - | - | - | - |
| 3907.43 | - | - | - | - | **+** | - | - |
| 3957.64 | - | - | - | **+** | - | - | - |
| 3982.93 | - | - | - | - | **+** | - | - |
| 4001.32 | - | - | - | - | **+** | - | - |
| 4034.08 | - | - | - | - | **+** | - | - |
| 4129.99 | - | - | **+** | - | **+** | - | - |
| 4215.48 | - | - | - | - | **+** | - | - |
| 4365.81 | - | **+** | - | - | - | - | - |
| 4491.23 | - | - | - | **+** | **+** | - | - |
| 4569.08 | **+** | - | - | - | - | - | **+** |
| 4585.51 | - | - | - | **+** | - | - | **+** |
| 4604.02 | - | - | - | - | - | - | **+** |
| 4648.64 | - | - | **+** | **+** | - | - | - |
| 4682.45 | - | - | - | **+** | - | - | - |
| 4856.07 | - | - | - | - | **+** | - | **+** |
| 4923.58 | - | - | - | - | - | - | **+** |
| 5071.34 | - | - | **+** | - | - | - | - |
| 5113 | - | - | **+** | - | - | - | - |
| 5165.87 | - | **+** | - | - | - | - | - |
| 5269.57 | - | - | - | - | - | **+** | - |
| 5346.15 | - | - | - | - | - | - | **+** |
| 5405.18 | - | - | - | - | - | - | **+** |
| 5586.55 | - | - | **+** | - | **+** | - | - |
| 5785 | - | - | - | **+** | - | - | - |
| 5920.52 | - | - | - | **+** | - | - | - |
| 6012.54 | - | - | - | - | - | **+** | - |
| 6312.27 | - | - | - | - | - | **+** | - |
| 6497.98 | - | - | - | - | - | **+** | - |
| 6518.89 | - | - | **+** | - | - | - | - |
| 6759.58 | - | **+** | - | - | - | - | - |
| 7030.66 | **+** | - | - | - | - | - | - |
| 7128.85 | **+** | - | - | - | - | - | - |
| 7287.72 | - | - | - | **+** | - | - | - |
| 7323.07 | - | - | - | - | **+** | - | - |
| 7380.2 | - | - | - | **+** | - | - | - |
| 7515.34 | **+** | - | - | - | - | - | - |
| 7817.42 | - | - | - | - | **+** | - | - |
| 7902.35 | - | - | - | **+** | - | - | - |
| 8035.36 | - | - | - | **+** | - | - | - |
| 8524.4 | - | **+** | - | - | - | - | - |
| 8658.57 | - | - | - | - | **+** | - | - |
| 8930.23 | - | - | - | - | **+** | - | - |
| 9127.56 | - | - | **+** | - | - | - | **+** |
| 9350.17 | - | - | - | **+** | - | - | - |
| 9503 | - | - | **+** | - | - | - | - |
| 9871.02 | - | - | - | - | - | **+** | - |
| 10123.31 | - | **+** | - | - | - | - | - |
| 10464.66 | - | - | - | **+** | - | - | - |
| 11661.05 | - | - | - | - | **+** | - | - |
| 12617.27 | - | - | - | - | - | **+** | - |
| 18998.15 | **+** | - | - | - | - | - | - |
| **Total** | **10** | **9** | **10** | **20** | **17** | **8** | **14** |

*List included uniquely species specific mass peaks. Da, Daltons; m/z, mass to charge.
